# Phenotype assessment for neurodegenerative murine models with ataxia and application to Niemann–Pick disease, type C1

**DOI:** 10.1242/bio.059052

**Published:** 2022-05-03

**Authors:** Julia Yerger, Antony C. Cougnoux, Craig B. Abbott, Rachel Luke, Tannia S. Clark, Niamh X. Cawley, Forbes D. Porter, Cristin D. Davidson

**Affiliations:** 1 Eunice Kennedy Shriver National Institute of Child Health and Human Development, Section on Molecular Dysmorphology, NIH, Bethesda, MD, 20892, USA; 2 National Human Genome Research Institute, Genetic Disease Research Branch, NIH, Bethesda, MD 20892, USA

**Keywords:** Niemann–Pick disease, Type C, Cerebellar ataxia, NPC1, Neurological disease, Phenotype assessment, Lysosomal disease

## Abstract

Identifying meaningful predictors of therapeutic efficacy from preclinical studies is challenging. However, clinical manifestations occurring in both patients and mammalian models offer significant translational value. Many neurological disorders, including inherited, metabolic Niemann–Pick disease, type C (NPC), exhibit ataxia. Both individuals with NPC and murine models manifest ataxia, and investigational therapies impacting this phenotype in mice have been reported to slow disease progression in patients (e.g. miglustat, intrathecal 2-hydroxypropyl-beta-cyclodextrin, and acetyl-_L_-leucine). Reproducible phenotypic scoring of animal models can facilitate comparisons between genotypes, sexes, disease course, and therapies. Previously, other groups have developed a composite phenotypic scoring system (CPSS), which was subsequently used to distinguish strain-dependent phenotypes and, with modifications, to evaluate potential therapies. However, high inter-rater reliability is paramount to widespread use. We have created a comprehensive, easy-to-follow phenotypic assessment based on the CPSS and have verified its reproducibility using murine models of NPC disease. Application of this scoring system is not limited to NPC disease and may be applicable to other models of neurodegeneration exhibiting motor incoordination, thereby increasing its utility in translational studies.

## Abstract (precis)

**Summary:** We present a detailed, comprehensive scoring system for murine models of NPC, and likely other neurodegenerative diseases, presenting with motor incoordination. This easy, relatively quick assessment yields high inter-rater reliability.

## INTRODUCTION

Phenotypic scoring systems for animal models of disease provide insight into disease progression and allow comparison of phenotypes between genotypes, treatment groups, strains, and sexes. In the context of Niemann–Pick disease, type C (NPC), and other forms of ataxia, phenotypic scoring focuses on evaluation of motor coordination and overall health status and represents an important readout measure for preclinical studies investigating potential therapeutic compounds.

While there are numerous behavioral readout measures as expertly reviewed by (Sukoff Rizzo and Crawley, 2017) for neurodegenerative disorders, most require specialized, often expensive, equipment, substantial time commitment from evaluators, and a dedicated behavioral space. Some common motor function assays for neurodegenerative diseases include the rotorod, open field, catwalk and footprint gait assessment. While the aforementioned assays provide valuable information, rapid, reproducible, cost-efficient options are quite limited. We propose one such assay using NPC disease as the model system and a phenotype scoring system originally developed by (Guyenet et al., 2010) as the foundation.

NPC is a rare, genetic disease with peripheral and neurodegenerative components. Clinical manifestations in NPC patients include but are not limited to cerebellar ataxia, hepatosplenomegaly, seizures, supranuclear gaze palsy, and cognitive impairment (Vanier, 2010). The transmembrane protein NPC1 functions in conjunction with soluble NPC2 protein to transport unesterified cholesterol out of the endolysosomal compartment. Pathogenic variants in *NPC1* (95% of cases) or *NPC2* (5% of cases) result in a decrease or loss of functional protein and ensuing accumulation of unesterified cholesterol and other lipids in the endolysosome (Vanier, 2010). Phenotypic manifestations in *Npc1^−/−^* mice, namely impairment of motor coordination, make mouse models of the disease well suited to a phenotypic scoring system.

A noticeable uptick in therapeutic development has occurred in the NPC research community over the past several years. Many biotechnology and pharmaceutical companies have joined the pursuit of academic investigators in the search for disease-modifying compounds for NPC patients. Importantly, several therapies with preclinical data demonstrating significant impact on disease phenotype (e.g. ataxia) have translated into clinical benefit for NPC patients (Zervas et al., 2001; Patterson et al., 2020a,b; Davidson et al., 2009; Ory et al., 2017; Kaya et al., 2021; Fields et al., 2021). However, meaningful comparisons between preclinical murine studies can be difficult given the number of behavioral assessments employed and the number of NPC1 murine models available. We present a rapid, easily adoptable scoring system that would facilitate more meaningful comparisons, both for independent studies using the same NPC1 murine model and for those originating from the same laboratory over the course of years.

Guyenet et al. developed a valuable composite phenotypic scoring system (CPSS) in 2010 to describe mouse models of cerebellar ataxia measuring: ledge test, hindlimb clasping, gait, and kyphosis (Guyenet et al., 2010). This scoring system was used to distinguish strain-dependent phenotypes in *Lyst*-mutant mice (Hedberg-Buenz et al., 2019), while a modified version was used in the context of NPC (Alam et al., 2016). A comparison between the different scoring systems is provided in Table 1. The applicability of these scoring systems, however, is limited by consistency errors between different individuals scoring genotypically identical mice. A specific, comprehensive, easily adoptable scale will allow evaluators to produce consistent and comparable results for phenotypic scoring of NPC and likely other models exhibiting ataxia.

**Table 1.**
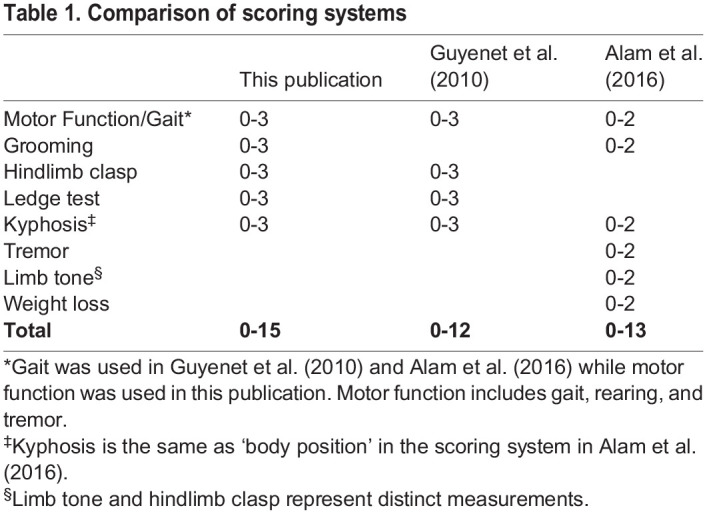
Comparison of scoring systems

We present a scoring system based on the CPSS developed by Guyenet et al. (2010). The method accurately depicts the neurological phenotype present in the different mouse models of NPC: hindlimb clasping, grooming, motor function, kyphosis, and ledge test. Each parameter is scored on a scale of 0 to 3, with 0 representing a phenotypically normal, healthy mouse with no observable deficits, and 3 representing the most severe form of that phenotype, which can help define humane endpoints for an institutional animal care and use committee (IACUC). For the purpose of demonstrating this phenotypic scoring system, we use Niemann–Pick disease, type C1 (NPC1) mice from two separate models. *Npc1^m1N^* and *Npc1^tm(I1061T)Dso^* mutant and *Npc1^+/+^* mice were generated by crossing heterozygous breeders, respectively (Loftus et al., 1997; Praggastis et al., 2015). The *Npc1^m1N^*-null model harbors a spontaneous frameshift mutation resulting in truncation of 11 of the 13 transmembrane domains of NPC1 (Loftus et al., 1997). As a result, *Npc1^m1N/m1N^* mice have a rapidly progressing disease, with onset of ataxia occurring at ∼6 weeks of age and death by ∼10 weeks of age. The *Npc1^tm(I1061T)Dso^* model was generated by knocking-in a missense mutation to recapitulate the most prevalent pathological variant found in NPC1 patients, p.I1061T (Praggastis et al., 2015). With a misfolded NPC1 protein targeted for endoplasmic-reticulum-associated degradation, this model exhibits a protracted disease course with onset of ataxia occurring at ∼8-9 weeks of age and death by ∼14 weeks of age. The phenotypic manifestations seen in *Npc1^I1061T/I1061T^* mice are delayed and marginally less severe than those observed in *Npc1^m1N/m1N^* mice.

Volunteers were provided with a description of the phenotype assay, along with written and visual examples of each score for the five different parameters. Subsequently, volunteers were asked to perform the phenotype score on a new set of videos or images for each parameter, devoid of any information to maintain blinded conditions. Inter-rater reliability (IRR) was then assessed to determine the reproducibility of the phenotype score for nominal and ordinal categories using Krippendorff's alpha (Hayes and Krippendorff, 2007; Krippendorff, 2011). Krippendorff's alpha is a kappa-like statistic that corrects for chance agreement and offers several advantages over other indices of IRR (Krippendorff, 2004). It can be used for two or more raters and categories; nominal, ordinal, interval, or ratio scales of measurements; large and small sample sizes; and in the presence or absence of missing data. It is not biased by raters’ preference in their use of categories. A value of 1 indicates perfect agreement, and 0 indicates the absence of reliability. Although the theoretical distribution of Krippendorff's alpha is unknown, an empirical distribution can be obtained using the bootstrap approach from which confidence intervals for the point estimate can be constructed (Zapf et al., 2016; Krippendorff, 2016).

## RESULTS

The detailed scoring parameters outlined below assume that the evaluator has already prepared the testing room and materials according to details provided in the Materials and Methods. In brief, the mice should be habituated to the testing room for at least 30 min prior to initiating the phenotype scoring assay and a clean, plastic mouse cage should be used for each mouse. The same sized cage and the same sequential evaluation of parameters should be maintained between mice and in longitudinal studies.

Text in *italics* is taken verbatim from Guyenet et al. (2010) and details have been modified or added to more reliably differentiate between scores, as well as to accurately reflect the disease phenotype seen in NPC murine models.

### Hindlimb clasp

*Hindlimb clasping is a marker of disease progression in a number of mouse models of neurodegeneration, including certain cerebellar ataxias*. First, *grasp the tail near its base and lift the mouse clear of all surrounding objects.* Avoid holding the mouse too close to the cage or other large objects, as a mouse often assumes a clasped hindlimb position when trying to reach for the cage or object, and this is not an accurate display of disease-related hindlimb clasping. Then, *observe the hindlimb position for 10 s* to determine the duration of hindlimb retraction. For more clarity, observe only one hindlimb for a total of 10 s, and then the other for a total of 10 s. Retraction consists of hindlimbs completely bent inward towards the abdomen. Hindlimbs that simply move towards the midline but remain fully or partially extended, do not count towards score.

**Fig. 1.**
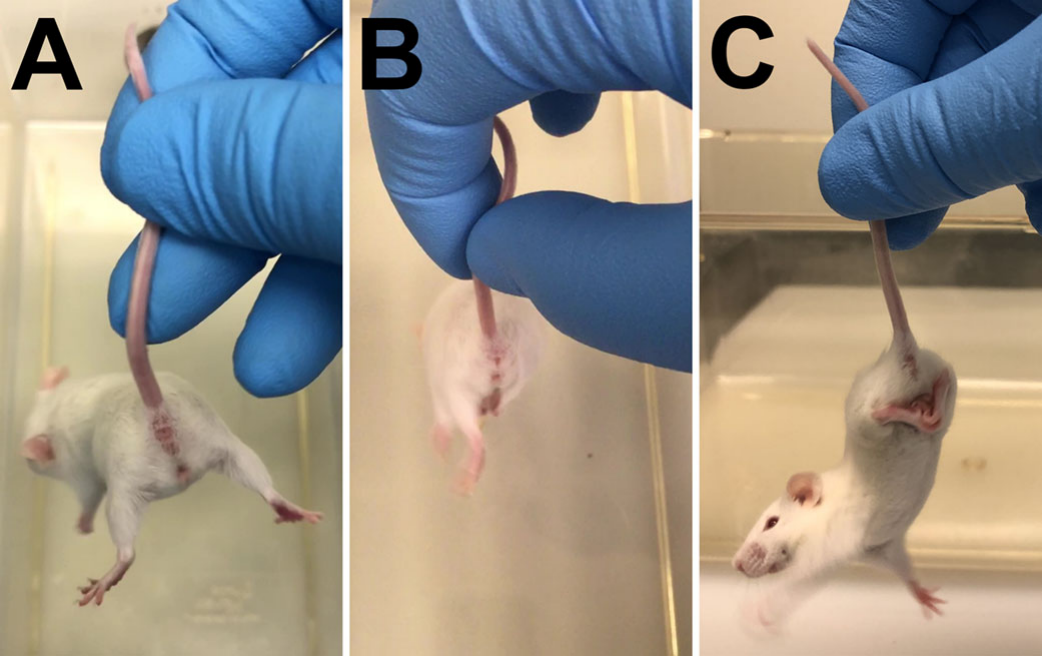
**Examples of limb retraction for evaluation of hindlimb clasping.** (A) Both limbs splayed out as seen with a score of 0. (B) One hindlimb (right) retracted towards the abdomen while the other (left) remains splayed out as seen with a score of 1 or 2, depending on how long either hindlimb is retracted. (C) Example of both hindlimbs toward and touching the abdomen, as seen with a score of 3.
(1) If hindlimbs individually, but not both at the same time, retract towards the abdomen for a cumulative time between 1 and 4 s (or less than 50% of the time of the test), the mouse is assigned a score of 1 (Fig. 1B).
(2) If hindlimbs individually, but not both at the same time, retract towards the abdomen for a cumulative time of 5 s or greater (or greater than 50% of the time of the test), the mouse is assigned a score of 2 (Fig. 1B).
(3) If both hindlimbs are retracted at the same time toward and touch the abdomen for any amount of time, the mouse is assigned a score of 3 (Fig. 1C).

(0) *If the hindlimbs are consistently splayed outward, away from the abdomen* and never retract towards the abdomen*,* the mouse is assigned a score of 0 (Fig. 1A).

Note that hindlimb clasping is not typically observed in the *Npc1^m1N^* mouse model, even in severely affected mutant mice. However, it may present with certain drug treatments or present more readily in different NPC mouse models such as *Npc1^tm1(I1061T)Dso^* and is therefore an important parameter.

The Krippendorff alpha point coefficient of agreement (bootstrap 95% CI) between observers using the scoring criteria for hindlimb clasp was 0.808 (0.319–1.00) as a nominal category and 0.907 (0.508–1.00) as an ordinal category. The nominal and ordinal point estimates suggest substantial and almost perfect agreement, respectively.

### Grooming

Grooming behavior maintains the appearance of the mouse's coat and is an indicator of disease progression and overall animal health. As mice lose motor coordination, they cannot self-groom as effectively as healthy mice (Kalueff et al., 2016). Moreover, as their overall health status continues to decline, less energy is devoted to grooming.

**Fig. 2.**
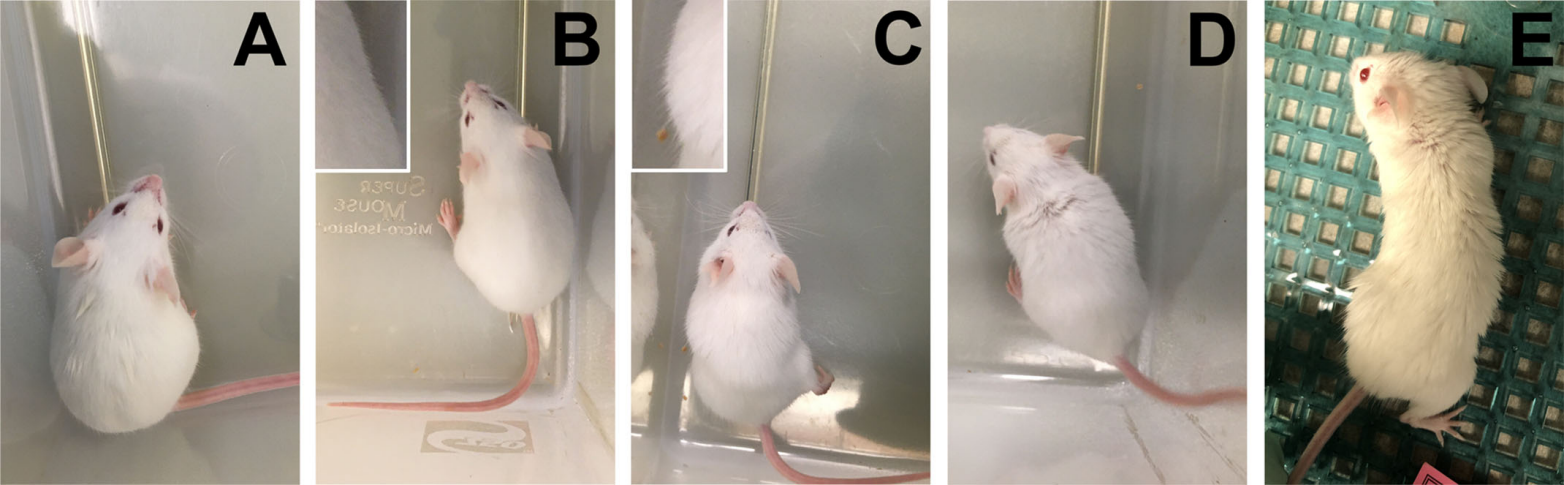
**Examples of grooming.** (A) Example of extraneous circumstance (wet fur) which does not count towards the grooming score. (B) score 0, (C) score 1, (D) score 2, and (E) score 3. Insets of (B) and (C) show normal versus piloerected fur, respectively.
(1) If a mouse has a mostly normal coat but piloerection is noticeable, it is assigned a score of 1 (Fig. 2C; inset highlights piloerection).
(2) If a mouse presents with small areas of uneven, patched, or thinned coat on the upper half of the body (head, shoulders, and upper back region), it is assigned a score of 2 (Fig. 2D). The roughed fur in this case is almost always piloerected.
(3) If a mouse displays a rough coat with uneven, patched, or thinned coat over most or all of its dorsal body (i.e. neck, shoulders, back, and hind end), it is assigned a score of 3 (Fig. 2E). Again, piloerection is almost always present.

Grooming scores should not be based on extraneous circumstances such as wet fur (Fig. 2A), fight wounds, or abnormalities from treatments such as ruffled fur or minor lesions/bald spots from barbering or repeated injections. Coat abnormalities at a local injection or surgery site are expected and do not reflect the health status of the animal but rather healing of wounded tissue. If possible, score the mouse's coat while omitting the extraneous circumstance. If clear delineation of the coat abnormality is not possible, omit score for this parameter/time point and note rationale for omission in data. Observe the mouse in the testing cage and note the appearance of its fur.

(0) If a mouse has a normal coat – i.e. smooth, shiny fur that does not have uneven patches, thinning or missing fur, or piloerection (standing up of fur) – it is assigned a score of 0 (Fig. 2B; inset demonstrates normal coat without piloerection).

The Krippendorff alpha point coefficient of agreement (bootstrap 95% CI) between observers using the scoring criteria for grooming was 0.692 (0.410–0.864) as a nominal category and 0.859 (0.567–0.958) as an ordinal category. The nominal and ordinal point estimates suggest substantial and almost perfect agreement, respectively.

### Motor function

Motor function *is a measure of coordination and muscle function.* For NPC disease, motor assessment includes both forward locomotion (emphasis placed on hindlimb splay, speed of movement, and tremor) and ability to rear. *Remove the mouse from its cage and place it* in the testing cage*.* Observe the mouse from above and from the side as it walks. Mice may be reluctant to move in the testing cage, but this should not be confused with inability to move. A gentle push or extra time to acclimate to the testing conditions should provide incentive to walk.

**Fig. 3.**
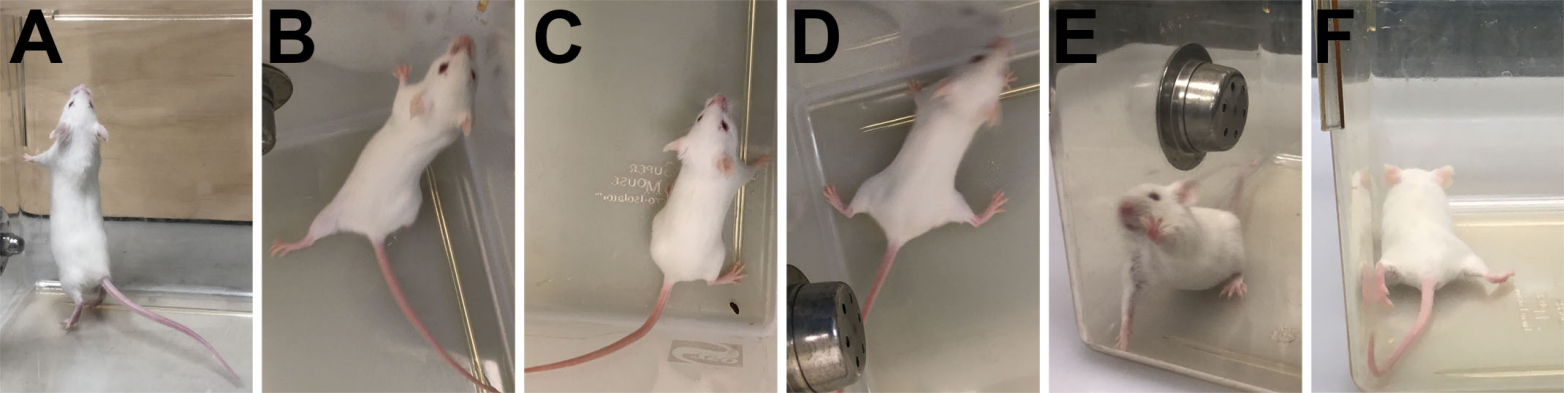
**Examples of hindlimb splay and rearing for evaluation of motor function.** (A) Normal, full height/extension during rear and (B) slippage of hindlimb during rear. (C) Mild hindlimb splay and (D) pronounced splay or ducked feet. (E) Attempted rear with hindlimbs starting farther away from cage side, thus preventing full normal upward extension and (F) abdomen dragging on ground with severe hindlimb splay.
(1) The mouse shows a slight resting *tremor or appears to limp* or wobble while walking. Uneven gait must be present for a score of 1, but the mouse may still move rapidly. Mouse is able to rear and achieve upward body extension, but hindlimbs typically slide backward during rear (Fig. 3B). Hindlimb splaying is often, but not always, observed (Fig. 3C). Mouse receives a score of 1.
(2) The mouse shows a moderate resting and moving tremor, *severe limp, lowered pelvis, or the feet point away from the body during locomotion (‘duck feet’)* (Fig. 3D). The mouse may move around the test cage but typically has slower movement. The mouse exhibits greater reluctance to rear and when rears are attempted, little upward extension is achieved, with hindlimbs sliding back upon initiation of and during rear (Fig. 3E). Mouse receives a score of 2.
(3) *The mouse has difficulty moving forward and drags its abdomen along the ground* (Fig. 3C). Severe tremor is present, movements are slower, hindlimbs drag along the walking surface, and the mouse may fall or roll over on its side during forward movement or rearing. Hind feet are consistently and noticeably splayed (pointed outward) during locomotion and even when stationary. Mouse is generally unable to rear and often will not try. If rears are attempted, the mouse will achieve very little height or will jump upward in an uncontrolled manner. Rears initiated by jumping most often result in immediate falling to the side and the mouse may appear to be in a hyper-excited state. Mouse receives a score of 3.

(0) *The mouse moves normally, with its body weight supported on all limbs, with its abdomen not touching the ground, and with both hindlimbs participating evenly*. Hindlimbs are in line with the body during the entirety of locomotion and are not splayed outward. Mouse moves quickly in a straight line with no difficulty turning. It is able to rear without issue, maintains hindlimb placement during the rear, and achieves full upward extension (Fig. 3A). Mouse receives a score of 0.

Note that repeated jumping which results in lateral falls is not normal and should not be interpreted as rearing.

The Krippendorff alpha point coefficient of agreement (bootstrap 95% CI) between observers using the scoring criteria for motor function was 0.750 (0.295–0.950) as a nominal category and 0.906 (0.399–0.998) as an ordinal category. The nominal and ordinal point estimates suggest substantial and almost perfect agreement, respectively.

### Kyphosis

**Fig. 4.**
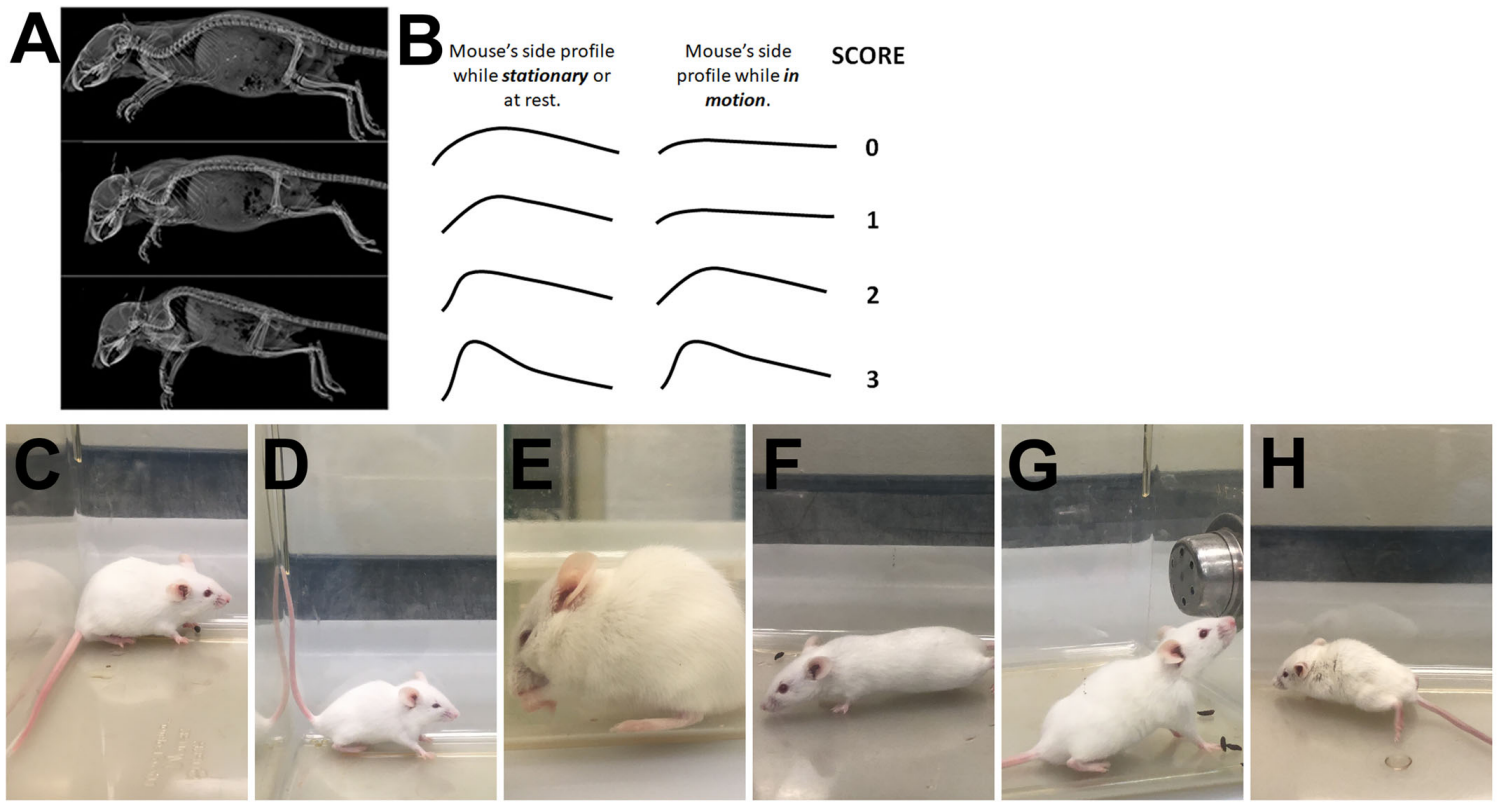
**Spine shapes to facilitate accurate evaluation of kyphosis and examples of kyphosis in stationary mice and during movement.** (A) X-ray images from Fig. 3 in Shazeeb et al. (2018) show the normal curvature of spine, mild kyphosis, and severe kyphosis (top, middle, and bottom panels, respectively) in a mouse model of achnodroplasia (Shazeeb et al., 2018). (B) Illustrations of a mouse's side profile while stationary (left side) and while walking (right side) and its associated kyphosis score. (C) Mild curvature of the back is frequently observed in normal mice at rest and receives a score of 0. A distinct, sharp curvature of the spine is observed with kyphosis as seen in moderate (D) and severe (E) cases. (D) would likely receive a score of 2 while (E) would likely receive a score of 3. (F) As seen with a score of 0 or 1, kyphosis is either absent or disappears during movement. As seen with a score of 2 and 3, mild (G) and severe (H) kyphosis is maintained during rearing and movement, respectively.
(1) If *the mouse exhibits mild kyphosis* when stationary (noticeable curvature of spine in upper back region; Fig. 4D), but at any time the mouse is *able to straighten its spine* (typically seen when walking; Fig. 4F), it is assigned a score of 1.
(2) If *the mouse is unable to straighten its spine completely and maintains persistent but mild kyphosis* even when walking, or if a mouse is never able to completely straighten its spine, it is assigned a score of 2 (Fig. 4D,G).
(3) If *the mouse maintains* obvious, *pronounced kyphosis* while stationary and while walking (Fig. 4E,H), it is assigned a score of 3.

*Kyphosis is a characteristic dorsal curvature of the spine that is a common manifestation of neurodegenerative disease in mouse models. It is caused by a loss of muscle tone in the spinal muscles secondary to neurodegeneration*. To better appreciate kyphosis, X-rays from Shazeeb et al. show mouse spines with different degrees of kyphosis (Shazeeb et al., 2018) (Fig. 4A; unaltered reproduction as permitted by https://creativecommons.org/licenses/by/4.0/legalcode) while drawings depict the basic curvature of spine for each score when mouse is at rest or in motion (Fig. 4B). Observe the mouse from the side of the cage (parallel to the walking surface) as it walks.

(0) *If the mouse is able to easily straighten its spine* during locomotion and while stationary, *and does not have persistent kyphosis*, it is assigned a score of 0 (Fig. 4C,F).

The Krippendorff alpha point coefficient of agreement (bootstrap 95% CI) between observers using the scoring criteria for kyphosis was 0.788 (0.337–1.00) as a nominal category and 0.923 (0.337–1.00) as an ordinal category. The nominal and ordinal point estimates suggest substantial and almost perfect agreement, respectively.

### Ledge test

*The ledge test is a direct measure of coordination, which is impaired in cerebellar ataxias and many other neurodegenerative disorders. This measure is the most directly comparable to human signs of cerebellar ataxia*. Of note, the ledge test brings together multiple aspects of movement, including balance, coordination, grip strength, and spatial awareness. *Observe the mouse as it walks along the cage ledge* and navigates the corners*.* Emphasis is placed on hindlimb engagement during forward motion and hind paw placement with respect to top or side of ledge. Gently place the mouse onto the long cage ledge, ensuring the hindlimbs can establish footing.

**Fig. 5.**
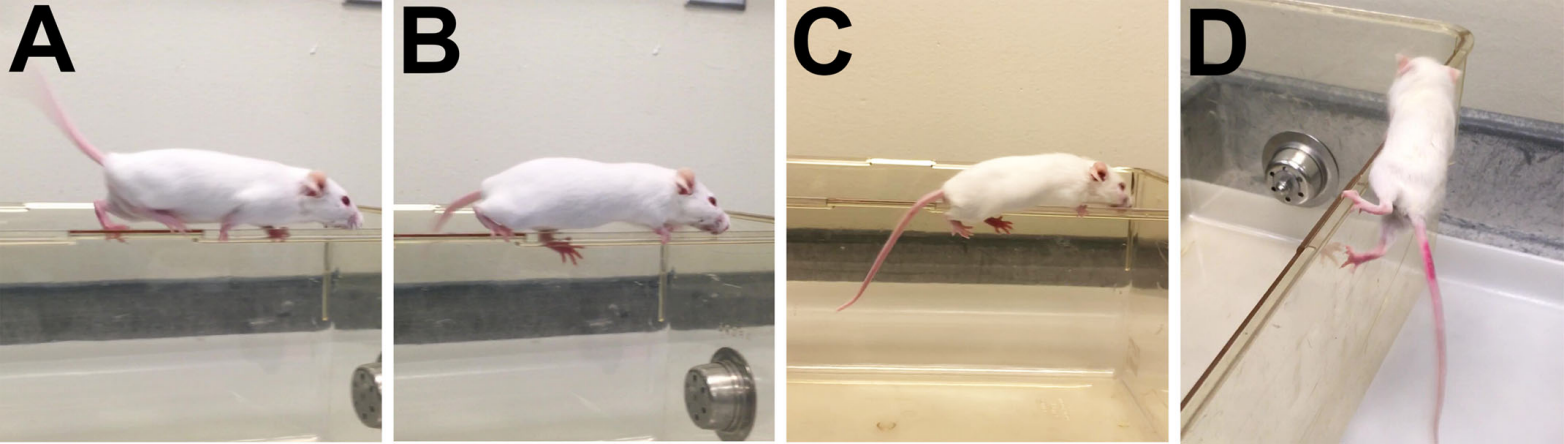
**Examples of solid and lost footing for evaluation of ledge test.** (A) Example of mouse with solid hindlimb footing during the ledge test where limbs are consistently placed high and near the cage edge, which receives a score of 0. (B) A mouse who has lost footing, or slips, during the ledge test will display feet below the cage edge. Slips occur when one, but not both, hindlimbs are on the side of the ledge. Lost footing is occasionally seen in a ledge score of 0 and is consistently seen in ledge score of 1. (C) A mouse with both hindlimbs hugging the sides of the cage and using them less or ineffectively would receive a score of 2 or 3, respectively. (D) A mouse that is unable to remain centered on the ledge which causes its hind end and hindlimbs to slip sideways off the ledge receives a score of 3.
(1) *The mouse loses its footing while walking along the ledge*, i.e. the hindlimbs repeatedly slip off the ledge (Fig. 5B) and mouse is less coordinated. Hindlimbs are still actively engaged in forward movement and mouse is able to walk with hindlimbs on top of the ledge for some portion of the traverse. The mouse can still navigate the corners, although it may struggle to do so. The mouse receives a score of 1.
(2) The mouse can move across the ledge, but it *does not effectively use its* hindlimbs as they are consistently hugging the sides of the ledge, rather than walking on top (Fig. 5C). Mouse is much less coordinated and when navigating corners, the mouse will often refuse to traverse the corner or its hindlimbs will fall off the ledge upon attempt. The mouse receives a score of 2.
(3) *The mouse falls off the ledge, or nearly so, while walking or attempting to lower itself, or shakes and refuses to move at all despite encouragement.* Although the mouse may be able to pull itself forward with hindlimbs hugging the sides of the ledge, it does so with great difficulty and without coordination. Mouse typically struggles to maintain an upright position on the ledge and will often fall to the side (Fig. 5D) and hang onto the ledge with only front paws. The mouse receives a score of 3.

(0) *A wild-type mouse will typically walk along the ledge* in a coordinated manner *without losing its balance* and will have consistent hindlimb footing placement on the top of the ledge (Fig. 5A). Mouse may have minor slips with normal coordinated recovery*.* The mouse will easily navigate the straight ledges and corners. Slight hesitation before entering a corner is normal, but once in motion, the mouse maintains coordination. The mouse receives a score of 0.

Note that *some mice will require a gentle nudge* to the hind end or gentle squeeze at the base of the tail *to encourage them to walk along the ledge or descend into the cage*. As mice age and become heavier (∼30+ grams), some will struggle with the ledge test. While this is typically not related to disease progression (unless obesity is a known disease phenotype), it still incurs a greater score on the ledge test.

The Krippendorff alpha point coefficient of agreement (bootstrap 95% CI) between observers using the scoring criteria for ledge test was 0.733 (0.549–0.860) as a nominal category and 0.899 (0.767–0.951) as an ordinal category. The nominal and ordinal point estimates suggest substantial and almost perfect agreement, respectively.

## DISCUSSION

Our adapted phenotypic scoring system is applicable to murine models of NPC1 and likely other models of neurological disorders with ataxic phenotypes. This scoring system can accurately distinguish between genotypes and track age-dependent disease progression, as demonstrated in the *Npc1^m1N^* model. Fig. S1 depicts the composite phenotype scores over time for three different mixed backgrounds of the *Npc1^m1N^* model (Fig. S1C, reproduced from Cawley et al., 2020). Additionally, Fig. S2A provides an example of average scores for each parameter over time for the BALB/cNctr-*Npc1^m1N^*/J model (Jackson Laboratory Stock number 003092). While some parameters seem to overlap, the phenotypic scores are not identical and thus supports inclusion of all five parameters to evaluate the phenotypic spectrum (Fig. S2B,C). This phenotypic scoring system provides a clinically relevant assessment for drug development in murine models and enhances confidence in translation of preclinical studies to anticipated impact on human disease.

The impetus for a more detailed adaptation of the CPSS by Guyenet et al. came from our experience using the CPSS and the variability we observed between different assessors (both new and seasoned), between studies done months to years apart, and between collaborating laboratories using the same NPC1 mouse models but housed in separate facilities. Understanding the importance and utility of a rapid assessment for large preclinical studies, we carefully considered the language for each scoring criterium and carried out two separate IRR assessments to improve reliability of the scoring. The ledge test required a third IRR assessment to achieve sufficient reproducibility.

IRR results from 14 independent assessors (17 for the third ledge test assessment), many of whom had no prior experience with phenotypic assessments, highlight this assay's reproducibility by both experienced and novice evaluators. Reliability coefficients were estimated using Krippendorff's alpha treating the phenotype scores as both nominal and ordinal measurements. A nominal scale is one for which there are three or more levels that cannot be logically ordered and an ordinal scale is one for which the levels can be logically ordered (Kraemer, 1992). Ordinal scales can be analyzed as ordinal and nominal data. In our results the ordinal coefficients were on average 19% larger than the nominal coefficients. The smallest increase was 12% for hindlimb clasp and the largest increase was 24% for grooming. The larger magnitude of the ordinal coefficients reflects the additional information about the data that ordinal scales give compared to nominal scales and the difference in the level of strictness required of nominal scales for a pair of ratings to agree. Ordinal scales distinguish between categories and show the rank order of the measurements. Rater agreement on nominal scales requires ratings to be identical. Any discrepancy is disagreement. On ordinal scales non-identical ratings can be given partial credit toward agreement to the degree that two ratings are closer in rank than two other ratings (Kraemer, 1992; Krippendorff, 2011). Although nominal coefficients are smaller than ordinal coefficients, they are not less ‘truthful’, just more stringent. A more stringent definition of agreement can identify category levels that raters find harder to discriminate between and thus reveal a need for better category definitions or more rater training.

Multiple guidelines (e.g., Brennan and Silman, 1992; Krippendorff, 2004; Landis and Koch, 1977; Shrout, 1998) have been proposed as benchmarks for labeling ranges of values from 0 (no reliability) to 1 (perfect reliability). Some are more conservative than others (Hallgren, 2012). One of the most often-used guidelines is given by Landis and Koch (1977) even though they state the divisions are ‘clearly arbitrary’ (Landis and Koch, 1977). Their guideline is given in Table S1. Using their descriptions, the reliability of the five phenotype scores are substantial as nominal coefficients and very nearly perfect as ordinal coefficients. In addition to the point estimates, we computed the bootstrapped 95% confidence intervals. In general, the intervals were wide with some of the lower limits in the fair agreement range. Wide confidence intervals, however, are more a reflection of the small sample sizes used to construct the confidence intervals than lower inter-rater reliability. The reliability of any measurement procedure can be improved by summing or averaging replicate measures (Shrout, 1998).

Some methodologists criticize the use of guidelines for judging reliability coefficients and stress that the magnitude of the coefficients should be interpreted in terms of its effects on the proposed research or clinical use. For example, the size of the IRR reflects the degree to which unreliability affects bias or precision of estimation, and on the power of a statistical test (Kraemer, 1992). The magnitude of the IRR is an index of the inflation of variance due to unreliability and indicates the degree of loss of precision in estimating population parameters such as the population mean or correlation coefficient. The inflation of within-group variance indicates the degree of attenuation of the effect size for comparing two means in hypothesis testing, and thus the attenuation of power. For example, to achieve the same level of power of a two-sample *t*-test for a measure with IRR=1.0, a measure with IRR=0.5 would need twice as many subjects, and a measure with IRR=0.2 would need five times as many subjects. Emphasizing the costs associated with incorrect decision making, Krippendorff (2004) stressed that when life is at stake, the criteria for reliability must be set far higher than when merely supporting scholarly arguments.

As with any measure based upon subjective assessment, some variation is still observed in this scoring system. As such, consistent and sufficient training should be provided to each evaluator to maintain the most accurate and reliable results. Blind assessment of mice is preferred, but in later stages of NPC and other neurological diseases, the obvious phenotype of affected mice prevents a completely blind assessment. Nonetheless, the detail provided in these scoring criteria can produce consistent results.

## MATERIALS AND METHODS

### Animal models

All procedures for this study were performed according to the Institutional Animal Care and Use Committee protocols approved by the National Human Genome Research Institute (protocol G-94-7) and the *Eunice Kennedy Shriver* National Institute for Child Health and Human Development (protocol 21-002). Heterozygous mice (*Mus musculus*) from BALB/cNctr-*Npc1^m1N^/*J (Jackson Laboratory stock number 003092) or *Npc1^tm(I1061T)Dso^* (Jackson Laboratory stock number 027704) colonies were crossed to generate respective *Npc1^−/−^* mutants and *Npc1^+/+^* control littermates. Male and female mice ranging in age from 4 to 14 weeks old were used to capture the full phenotypic range of disease progression. There were no exclusion criteria set and mice were randomly selected for evaluation. Raters were blinded to age and genotype of mice.

### Tools and procedure for phenotype assessment

**Fig. 6.**
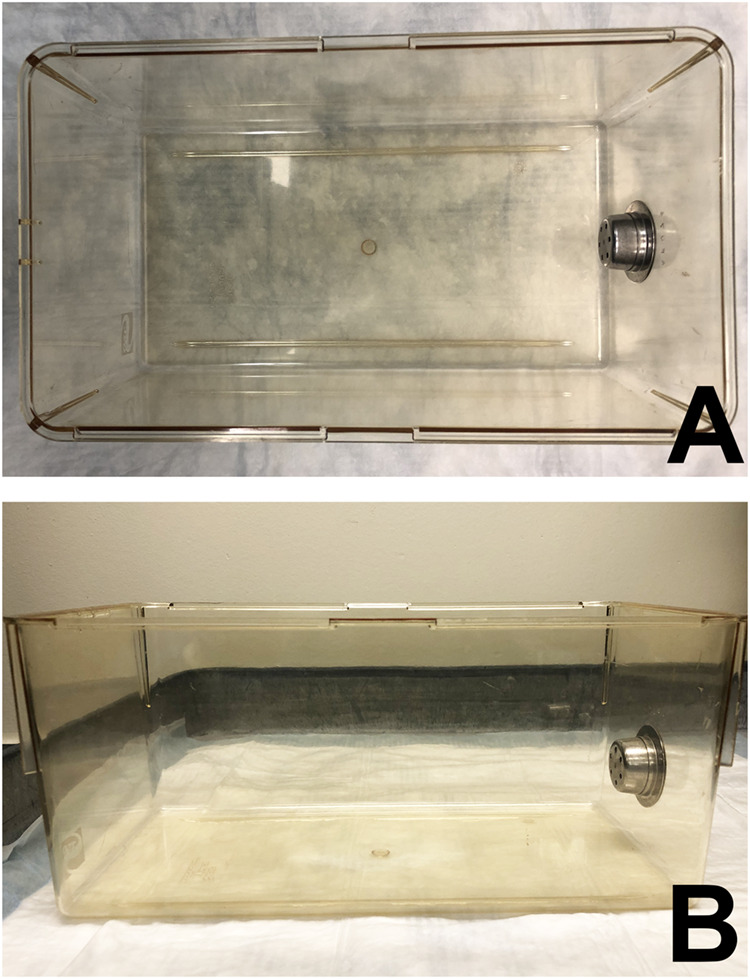
**Example of a cage used for the phenotypic assessment.** Top (A) and side (B) view of a Super Mouse micro-isolator cage bottom with dimensions of approximately 20 cm×34.5 cm×14.5 cm, four walkable ledges, and four navigable ledge corners.

A clear, plastic cage (approximately 20 cm×34.5 cm×14.5 cm) absent of bedding, food, water, and other mice is used for evaluation (Fig. 6). Although the material and cage dimensions are suggested, the most important factor is to maintain consistent cage choice for all mice evaluated for the duration of longitudinal studies. The mice must have enough space to walk freely. The cage must consist of ledges that a mouse can traverse, with navigable ledge corners. Video recording is optional but, if desired, filming is recommended for at least 30 s from a bird's eye view and at least 30 s parallel to the walking surface, as observed from the long side of the cage. The cage should be wiped with an odorless disinfectant (e.g. chlorine dioxide or isopropyl alcohol, Hershey et al., 2018) between animals, unless new cages are used for each mouse.

Bedding can be present in the testing cage if desired, but this makes evaluation of motor function more difficult, especially when observing ducked or splayed hindlimbs. Also, the experiment should be conducted in a room with as few environmental stressors as possible.

**Fig. 7.**
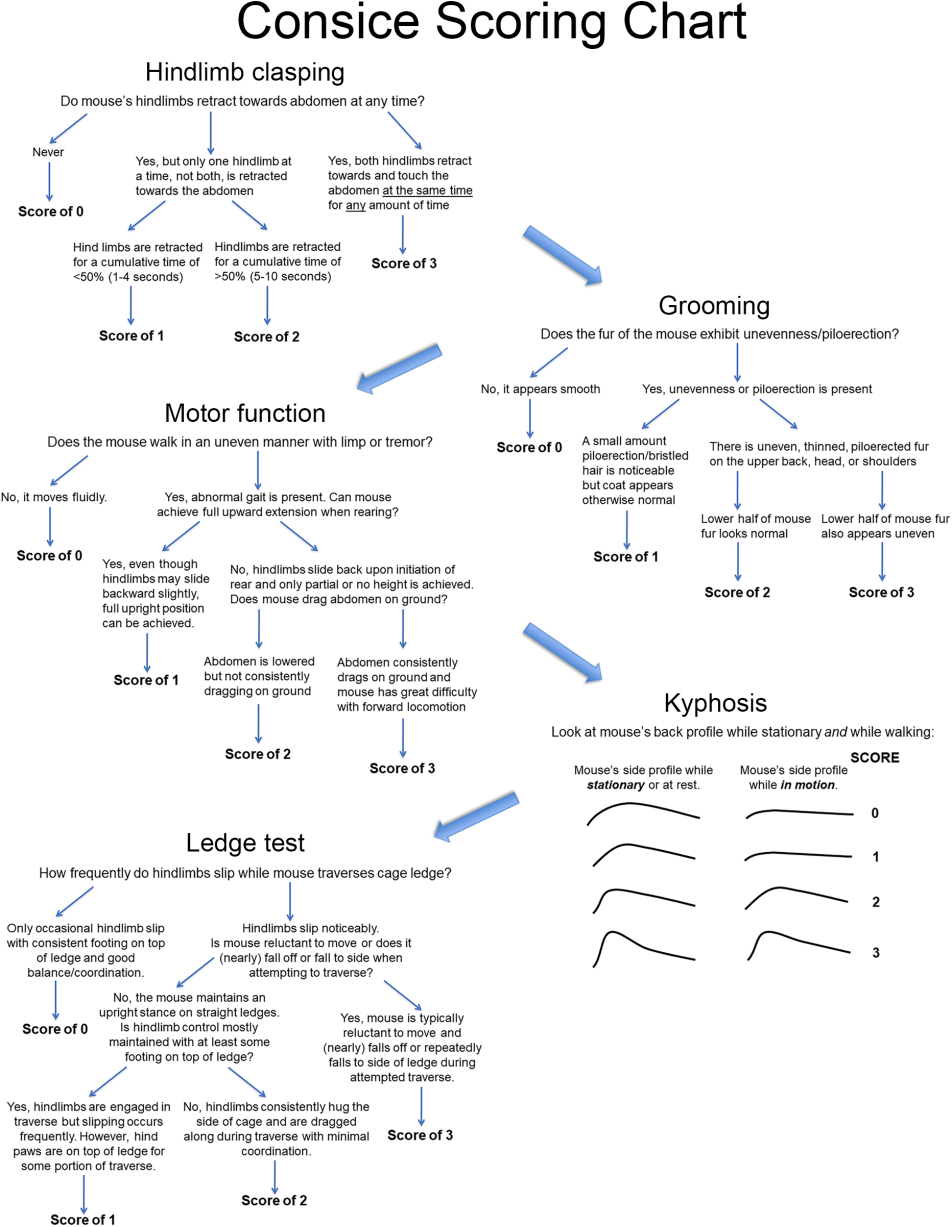
**Concise scoring chart.** Suggested flow chart for phenotype evaluation with abbreviated criteria for scores within each parameter.

Mice are transferred to the testing room and allowed to acclimatize for a minimum of 30 min, longer if conditions in the new room are different from their normal housing room. Playing the radio or white noise at a low volume is recommended during acclimatization and evaluation to minimize the mice's startle response from extraneous noises. A clean, dry testing cage or box should be obtained in accordance with the description provided above. Testing should always be done sequentially, with the following order recommended: hindlimb clasping, grooming, motor function, kyphosis, and finally ledge test. A concise scoring chart is provided for quick, easy reference to parameters and criteria for each score (Fig. 7). For *Npc1* mouse models, tests are suggested to be performed on the same day once per week starting at 4 weeks of age. Phenotype scoring can be highly variable before 5 weeks of age since mice are less focused (juvenile state). However, by starting earlier the animals become familiar with the procedure, which results in less stress during subsequent tests, thereby improving the reliability of results in later measurements. Also, depending on the genetic background, the *Npc1* mutant mice can be severely affected as early as 5 weeks of age (Parra et al., 2011). Given that mice are nocturnal and activity levels are known to fluctuate (Refinetti, 2006; Poffé et al., 2018), mice should always be tested within the same 2–3 h block of time (e.g. late morning, early afternoon, late afternoon). Also, because murine models of NPC disease typically have rapid progression, testing is suggested to be carried out at a minimum of every other week until mice reach the study or humane endpoint. Frequency and age of initiation of testing for other diseases may differ depending on how rapidly the disease progresses.

### Statistical analysis

Fourteen observers, including both experienced and novice phenotypic scorers, rated videos or images of mice on each parameter (except ledge test) using the descriptions described herein. Seventeen observers of mixed expertise were used for evaluation of the ledge test, again using descriptions provided. All observers scored five videos for motor function, kyphosis, and hindlimb clasp, 12 videos for ledge test, and seven still images for grooming. Scores were analyzed as nominal and ordinal categories using Krippendorff's alpha to verify the assay's reproducibility (Table S2). Analyses were conducted on the R statistical computing platform version 3.6.3, 2020-02-29 (Vienna, Austria; https://www.R-project.org/) using the Rstudio integrated development environment (IDE) Version 1.2.1335 (RStudio, Inc., Boston, MA, USA; http://www.rstudio.com/). Krippendorff's alpha and the 95% bootstrap confidence intervals were computed using the R-function K_alpha (Zapf et al., 2016). K_alpha was modeled after the R-function kripp.alpha from the IRR package and the SAS-macro kalpha from Andrew Hayes, and is available from Zapf et al., (2016). The bootstrap procedure used 1000 independent replications.

Nonparametric Spearman correlation between individual phenotype parameters was performed using GraphPad Prism version 9.0.0 for Windows, GraphPad Software, San Diego, CA, USA, www.graphpad.com.

## Supplementary Material

Supplementary information
