# Supplementary material for: Phenotype assessment for neurodegenerative murine models with ataxia and application to Niemann–Pick disease, type C1
Source: Biol Open. 2022 May 3;11(4):bio059052. doi: 10.1242/bio.059052 (PMC9096702; doi:10.1242/bio.059052)
Supplement: Supplementary information [file biolopen-11-059052-s1.pdf]

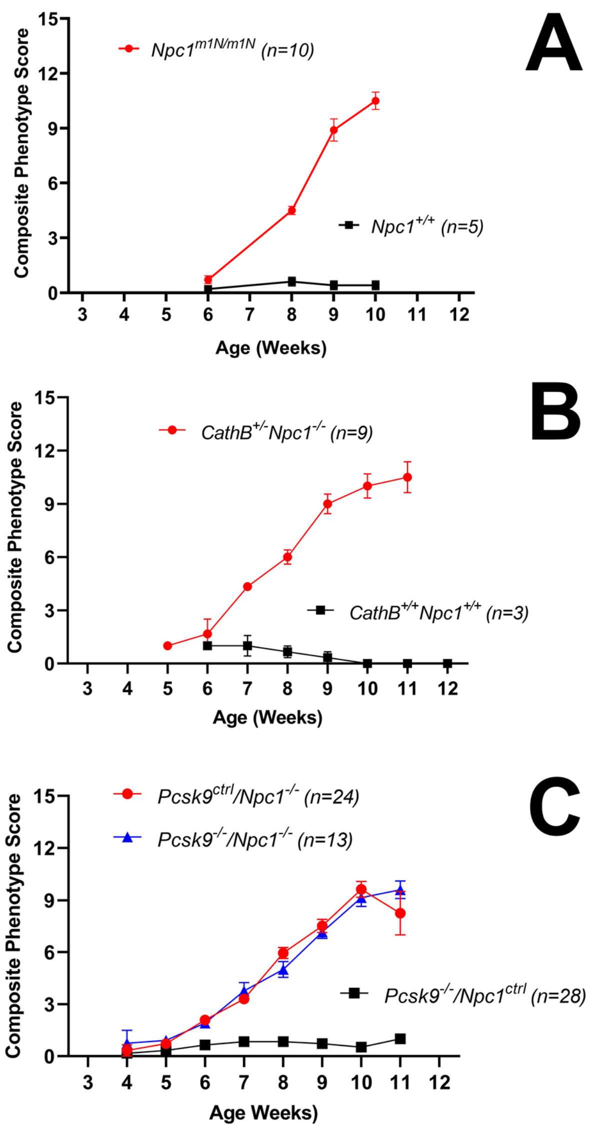

Fig. S1. Time course for *Npc1^{m1N}* model on different backgrounds. (A) Time course of composite phenotype scores for BALB/cNctr-*Npc1^{m1N}*/J mice (Jackson Laboratory Stock No. 003092) evaluated in laboratory A by one individual. (B) Time course of composite phenotype scores for C57Bl/6:SV129:BALB/c-*Npc1^{m1N}* evaluated in laboratory B by two individuals. (C) Time couse of composite phenotype scores for C57Bl/6:BALBc-*Npc1^{m1N}* mice evaluated in laboratory B by two individuals (previously published data with modifications to graph layout as permitted by the open access Creative Common CC BY license (Cawley et al., 2020)). Even with different mixed backgrounds, *Npc1^{m1N}* mice exhibit very similar phenotype scores as disease progresses. All values represent mean±s.e.m. for biological replicates.

Biology Open • Supplementary information

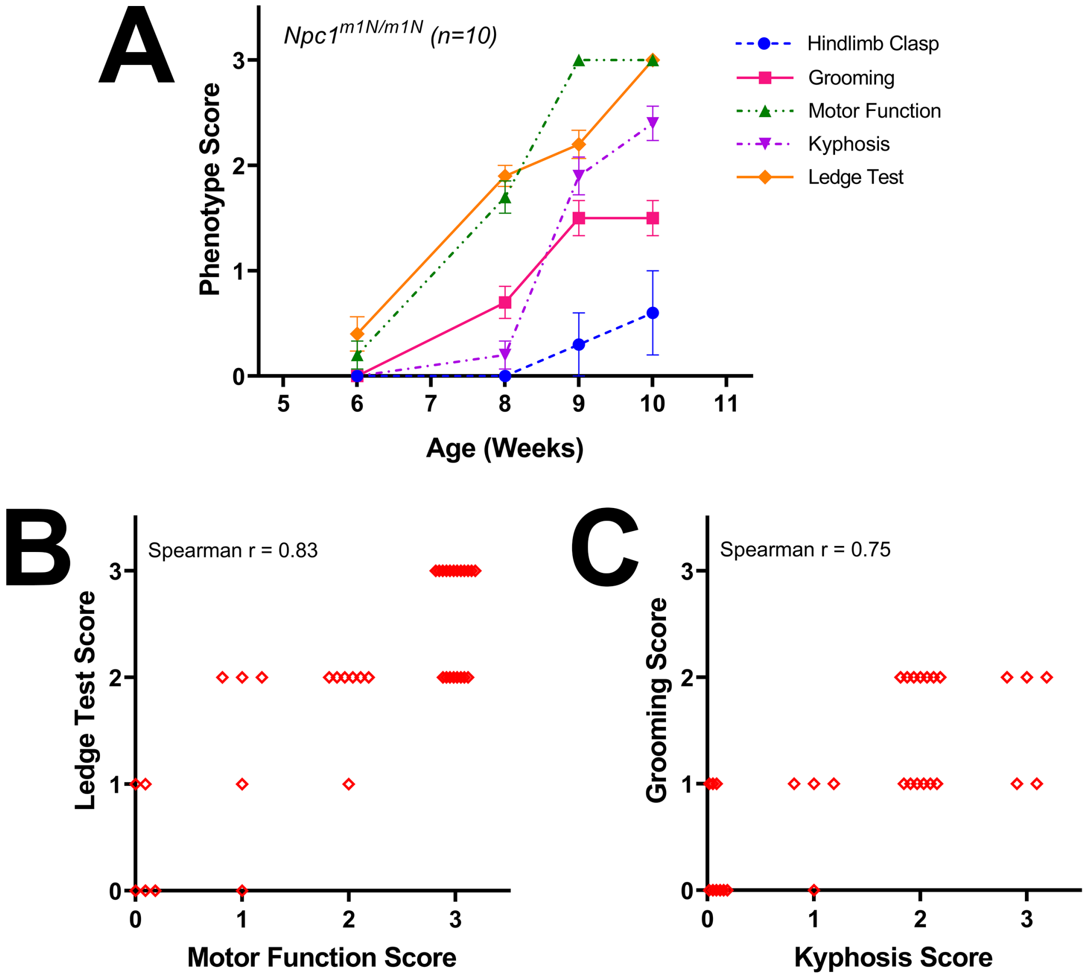

Fig. S2. Individual parameters over time and correlation between parameters. (A) Using the *Npc1*$^{m1N/m1N}$ mice depicted in Supplemental Fig. 1A as an example, the time course for each phenotype parameter is presented (n = 10). (B, C) Although some parameters seem to overlap, analysis of correlation suggest incomplete agreement for ledge test and motor function (B; Spearman's coefficient r = 0.83) as well as grooming and kyphosis (C; Spearman's coefficient r = 0.75). Values in (A) represent mean±s.e.m. for biological replicates. Values in (B, C) represent biological replicates over time (scores for mice depicted in (A) at 6, 8, 9, and 10 weeks of age).

Biology Open • Supplementary information

Table S1. Reliability Coefficient Guidelines (Landis & Koch, 1977)

kappa	Interpretation
0.00	Completely Random Agreement
0.01 – 0.20	Slight agreement
0.21 – 0.40	Fair agreement
0.41 – 0.60	Moderate agreement
0.61 – 0.80	Substantial agreement
0.81 – 1.00	Almost perfect to perfect agreement

Table S2. Krippendorff's alpha for ratings analyzed as nominal and ordinal categories

Parameter	Sample size	Krippendorff's alpha	
		nominal	ordinal
Hindlimb clasp	14 raters, 5 videos	0.808	0.907
Bootstrap 95% CI		0.319 – 1.000	0.508 – 1.000
Grooming	14 raters, 7 images	0.692	0.859
Bootstrap 95% CI		0.410 – 0.864	0.567 – 0.958
Motor function	14 raters, 5 videos	0.750	0.906
Bootstrap 95% CI		0.295 – 0.950	0.399 – 0.998
Kyphosis	14 raters, 5 videos	0.788	0.923
Bootstrap 95% CI		0.337 – 1.000	0.337 – 1.000
Ledge test	17 raters, 12 videos	0.733	0.899
Bootstrap 95% CI		0.549 – 0.860	0.767 – 0.951

Bootstrap confidence intervals computed using n = 1000 replications.

Biology Open • Supplementary information

References

CAWLEY, N. X., LYONS, A. T., ABEBE, D., WASSIF, C. A. & PORTER, F. D. 2020. Evaluation of the Potential Role of Proprotein Convertase Subtilisin/Kexin Type 9 (PCSK9) in Niemann-Pick Disease, Type C1. *Int J Mol Sci,* 21.
